# European Consensus on Malabsorption—UEG & SIGE, LGA, SPG, SRGH, CGS, ESPCG, EAGEN, ESPEN, and ESPGHAN. Part 1: Definitions, Clinical Phenotypes, and Diagnostic Testing for Malabsorption

**DOI:** 10.1002/ueg2.70012

**Published:** 2025-03-25

**Authors:** Marco Vincenzo Lenti, Heinz Florian Hammer, Ilja Tacheci, Rosa Burgos, Stephane Schneider, Anastasiou Foteini, Aleksejs Derovs, Jutta Keller, Ilse Broekaert, Marianna Arvanitakis, Dan Lucian Dumitrascu, Oscar Segarra‐Cantón, Željko Krznarić, Juris Pokrotnieks, Gonçalo Nunes, Johann Hammer, Loris Pironi, Marc Sonyi, Cristina Maria Sabo, Juan Mendive, Adrien Nicolau, Jernej Dolinsek, Denisa Kyselova, Lucrezia Laterza, Antonio Gasbarrini, Teodora Surdea‐Blaga, Jorge Fonseca, Christos Lionis, Gino Roberto Corazza, Antonio Di Sabatino

**Affiliations:** ^1^ Department of Internal Medicine and Medical Therapeutics University of Pavia Pavia Italy; ^2^ First Department of Internal Medicine Fondazione IRCCS San Matteo Pavia Italy; ^3^ Division of Gastroenterology and Hepatology Department of Internal Medicine Medical University Graz Austria; ^4^ 2nd Department of Internal Medicine ‐ Gastroenterology Faculty of Medicine in Hradec Králové University Hospital Hradec Králové Charles University Hradec Kralove Czech Republic; ^5^ Endocrinology and Nutrition Department Hospital Universitari Vall d'Hebron Diabetes and Metabolism Research Unit Vall d'Hebron Institut de Recerca (VHIR) Universitat Autonoma de Barcelona Barcelona Spain; ^6^ Gastroenterology and Nutrition Centre Hospitalier Universitaire de Nice Université Côte d'Azur Nice France; ^7^ 4th Local Primary Care Team Municipality Practice and Academic Practice of Heraklion University of Crete Heraklion Greece; ^8^ Department of Internal Diseases Rīga Stradiņš University Riga Latvia; ^9^ Israelitic Hospital Academic Hospital University of Hamburg Hamburg Germany; ^10^ Department of Paediatrics Faculty of Medicine and University Hospital Cologne University of Cologne Cologne Germany; ^11^ Department of Gastroenterology Digestive Oncology and Hepatopancreatology HUB Hôpital Erasme Université Libre de Bruxelles Brussels Belgium; ^12^ 2nd Department of Internal Medicine Iuliu Hatieganu University of Medicine and Pharmacy Cluj‐Napoca Romania; ^13^ 2nd Medical Department Emergency Clinical County Hospital Cluj‐Napoca Romania; ^14^ Paediatric Gastroenterology and Clinical Nutrition Unit Vall d'Hebron Hospital Universitari Vall d'Hebron Barcelona Hospital Campus Barcelona Spain; ^15^ Department of Gastroenterology, Hepatology and Nutrition University of Zagreb Zagreb School of Medicine University Hospital Centre Zagreb Zagreb Croatia; ^16^ Centre of Gastroenterology Hepatology and Nutrition Pauls Stradiņš Clinical University Hospital Riga Latvia; ^17^ Gastroenterology Department Hospital Garcia de Orta Almada Portugal; ^18^ Egas Moniz Center for Interdisciplinary Research (CiiEM) Egas Moniz School of Health & Science Almada Portugal; ^19^ Department of Gastroenterology and Hepatology Medical University of Vienna Vienna Austria; ^20^ Department of Medical and Surgical Sciences University of Bologna Bologna Italy; ^21^ Centre for Chronic Intestinal Failure IRCCS Azienda Ospedaliero‐Universitaria di Bologna Bologna Italy; ^22^ Clinic for General Medicine, Gastroenterology, and Infectious Diseases Augustinerinnen Hospital Cologne Germany; ^23^ La Mina Primary Health Care Academic Centre Catalan Health Institute University of Barcelona Barcelona Spain; ^24^ Pediatric Gastroenterology Hepatology and Nutrition Unit Pediatric Department University Medical Center Maribor Maribor Slovenia; ^25^ Faculty of Medicine University of Maribor Maribor Slovenia; ^26^ Department of Hepatogastroenterology IKEM Prague Czech Republic; ^27^ Department of Translational Medicine and Surgery Università Cattolica del Sacro Cuore Rome Italy; ^28^ CEMAD Fondazione Policlinico Universitario A. Gemelli IRCCS Rome Italy; ^29^ Laboratory of Health and Society School of Medicine University of Crete Heraklion Greece

**Keywords:** breath test, coeliac disease, diarrhoea, enteropathy, nutrition, pancreatitis, weight loss

## Abstract

Malabsorption is a complex and multifaceted condition characterised by the defective passage of nutrients into the blood and lymphatic streams. Several congenital or acquired disorders may cause either selective or global malabsorption in both children and adults, such as cystic fibrosis, exocrine pancreatic insufficiency (EPI), coeliac disease (CD) and other enteropathies, lactase deficiency, small intestinal bacterial overgrowth (SIBO), autoimmune atrophic gastritis, Crohn's disease, and gastric or small bowel resections. Early recognition of malabsorption is key for tailoring a proper diagnostic work‐up for identifying the cause of malabsorption. A patient's medical and pharmacological history is essential for identifying risk factors. Several examinations such as endoscopy with small intestinal biopsies, non‐invasive functional tests and radiological imaging are useful in diagnosing malabsorption. Because of its high prevalence, CD should always be looked for in cases of malabsorption with no other obvious explanations and in high‐risk individuals. Nutritional support is key in the management of patients with malabsorption; different options are available, including oral supplements, enteral or parenteral nutrition. In patients with short bowel syndrome, teduglutide proved effective in reducing the need for parenteral nutrition, thus improving the quality of life of these patients. Primary care physicians play a central role in the early detection of malabsorption and should be involved in multidisciplinary teams for improving the overall management of these patients. In this European consensus, involving ten scientific societies and several experts, we have dissected all the issues around malabsorption, including the definitions and diagnostic testing (Part 1), high‐risk categories and special populations, nutritional assessment and management, and primary care perspective (Part 2).

## 1 Introduction

Malabsorption is a complex syndrome, with multifold clinical manifestations, characterised by the defective passage of one (i.e., selective malabsorption) or more (i.e., partial or global malabsorption) nutrients through the intestinal mucosa to the blood/lymphatic stream [1, 2]. Since the mechanisms of absorption are numerous and multifaceted, involving several parts of the gastrointestinal tract (i.e., stomach, liver, pancreas, small bowel, large bowel), the detailed understanding of malabsorption and its causes has evolved slowly, and has been fuelled by the progresses made on coeliac disease (CD), which has paved the way to the study of small bowel abnormalities and other enteropathies (Table 1). Indeed, over the last decades, the attention has shifted from malabsorption syndrome to the diseases causing malabsorption. For this reason, several malabsorption tests (e.g., triglyceride test, oxalate loading test, ^14^C triolein breath test, Schilling test) and unspecific enterocytic damage markers (e.g., intestinal fatty acid binding protein, diamine oxidase, citrulline), burdened by difficult interpretability, high costs, and in some cases exposure to radioisotopes [2], have either never entered into clinical practice or have been rapidly abandoned in favour of more precise, disease‐specific, testing such as CD serology. Clinically, diarrhoea and steatorrhoea have been considered the cornerstone manifestations of malabsorption [1], though are just a part of the wide spectrum of clinical presentations ranging from being asymptomatic to systemic involvement. Therefore, the most difficult challenge for any physician is to raise the suspicion of malabsorption, which in the first instance derives from the patient's clinical history. For all the abovementioned reasons, it is not surprising that despite the relevance of malabsorption, both in terms of its epidemiology and clinical implications, no formal guideline or consensus on this topic has ever been published. Indeed, several disease‐ or symptom‐based guidelines exist but none of those specifically deal with malabsorption.

**TABLE 1:** List of conditions causing enteropathy that may be responsible for generalised malabsorption syndrome and their main features.

Conditions causing enteropathy	Main features
Coeliac disease (CD; also including seronegative CD, ulcerative jejuno‐ileitis, refractory CD type I and II, enteropathy‐associated T‐cell lymphoma, dermatitis herpetiformis)	HLA‐restricted, gluten‐sensitive villous atrophy; positive coeliac serology in most cases
Collagenous sprue	Significant small bowel sub‐epithelial collagen band; often associated to CD or other enteropathies (e.g., olmesartan enteropathy)
Autoimmune enteropathy	Paediatric or adult onset; often associated to other autoimmune disorders; anti‐enterocyte antibodies positivity
Iatrogenic enteropathies	Secondary to olmesartan & other angiotensin II receptor blockers, mycophenolate mofetil, chemotherapy, radiotherapy, checkpoint inhibitors, graft‐versus‐host disease, transplanted small intestine, small bowel radiation
Common variable immune deficiency	Deficient serum IgA, IgG, and IgM, greater likelihood of infections, autoimmunity, and cancers; absence of plasma cells in duodenal biopsies
Crohn's disease	Chronic inflammatory disorder that may affect the whole gastrointestinal tract with skip, deep, transmural, ulcers; granulomas may be present
Tropical sprue	Villous atrophy in patients living in poor hygienic conditions, especially in rural areas of developing countries; incidence progressively decreased with improvement of hygienic conditions
Eosinophilic enteropathy	Eosinophilic infiltration of the small bowel mucosa, may be associated with other Th2 disorders or a more extensive gastrointestinal eosinophilic involvement
Food protein‐induced enterocolitis	Rare, cell‐mediated, food hypersensitivity affecting infants, causing severe diarrhoea with villous atrophy, dehydration and sepsis‐like picture after the ingestion of cow's milk, soy, or other food proteins
Protein energy malnutrition	Usually determines partial villous atrophy in severely starved patients
Small intestinal bacterial overgrowth	Occurring in patients with predisposing conditions; may rarely cause villous atrophy
Indolent CD4+ T‐cell lymphoma	Slow‐progressing non‐Hodgkin lymphoma
HIV enteropathy	Villus atrophy occurring in HIV‐infected patients; may cause severe weight loss and ‘wasting syndrome’; usually recovers with anti‐retroviral therapy
Giardiasis	Parasitic infection that may become chronic, causing villous atrophy and diarrhoea; the diagnosis relies on searching *G. lamblia* in faeces, duodenal aspirate or duodenal biopsy
Whipple's disease	Disease caused by *Tropheryma whipplei*, typically affecting middle‐aged male individuals; usually onset with arthritis, may cause systemic involvement; PAS + macrophages infiltrating the lamina propria of the duodenum
Idiopathic/unclassified villous atrophy	Villous atrophy that does not follow under any of the aforementioned conditions; unknown causes of villous atrophy

As it appears evident in the literature, some confusion exists with regard to the use of the terms maldigestion, malabsorption, and malassimilation, sometimes used interchangeably, making it even more difficult to deal with this clinical issue. The challenges around malabsorption are also constantly changing and evolving [3]; suffice it to say that some causes of malabsorption that were common in the last century, such as tropical sprue and small bowel tuberculosis, have mostly disappeared, while others, such as some drug‐induced enteropathies, CD, or Crohn's disease, are on the rise, as discussed later. Hence, we herein propose the first European consensus on malabsorption, which was developed by ten European scientific societies and thirty experts in the field, in order to define malabsorption and malabsorption syndromes, suggest the tests for making a diagnosis, identify the populations at risk of malabsorption, and discuss the overall management. The consensus will not focus on the diagnostic and management aspects of single diseases.

## 2 Materials and Methods

### 2.1 General Framework

Each participating Scientific Society (Società Italiana di Gastroenterologia [SIGE], Latvian Gastroenterologists Association [LGA], Sociedade Portuguesa de Gastrenterologia [SPG], Societatea Română de Gastroenterologie şi Hepatologie [SRGH], Czech Society of Gastroenterology [CGS], European Society for Primary Care Gastroenterology [ESPCG], European Association for Gastroenterology, Endoscopy and Nutrition [EAGEN], The European Society for Clinical Nutrition and Metabolism [ESPEN], and the European Society for Paediatric Gastroenterology, Hepatology and Nutrition [ESPGHAN]) nominated representatives to take part to the drafting of the consensus, and the United European Gastroenterology (UEG) provided finalcial and technical support. Four distinct working groups, made up of five to seven experts of the field, were selected after the first online meeting held in November 2021. Thereafter, in June 2022, a fifth group of primary care physicians with expertise in gastrointestinal disorders also joined. A group leader coordinated the steps in each group. Three main research questions were assigned to each working group for a total of 11 areas including the identification, diagnostic, screening and management issues of malabsorption. The specific framework for performing the systematic review was drafted by the consensus coordinator (MVL) and sent to all the participants by email. The PRISMA recommendations for systematic analysis of the literature were followed [4], and a 2020 PRISMA flow diagram was filled in for the research questions, when appropriate, by using specific Medical Subject Heading terms (MeSH; Supporting Information [Link], [Link], [Link], [Link], [Link], [Link]).

The four working groups focussed on the following topics: Group 1—Definitions, clinical phenotypes, and diagnostic criteria of malabsorption; Group 2—Initial assessment, testing for malabsorption and its causes; Group 3—Screening for malabsorption and special populations; Group 4—Nutritional supplementation, treatment goals, and supportive care. Finally, Group 5 provided the primary care perspective in each of the previous groups. The consensus coordinators (MVL, ADS, GRC) harmonised and supervised each step. Multiple online and face‐to‐face meetings were held during the entire period needed to complete the consensus (November 2021–June 2024). All participants met yearly for reporting on the progress of the consensus at the annual UEG Week, either in presence or online.

### 2.2 Step 1: Literature Search and Questions

The online platforms Pubmed and EMBASE were used for literature search in September 2022–April 2023, by using standardised MeSH terms with the connectors ‘OR’, ‘AND’. The search was not restricted to the title or the abstract but all papers were considered. Each working group searched for all papers published since database inception (i.e., with no temporal restrictions) and written (or translated) in English. Additionally, we searched the reference lists of landmark reviews or systematic reviews dealing with the specific issue of the research question. The research questions were those described above, and the first literature review focussed on those questions. In the end, each working group filled in several PRISMA flow diagrams (Supporting Information [Link], [Link], [Link], [Link], [Link], [Link]) for choosing the relevant papers to be included. Since thousands of papers were identified, in the explanatory text, only the most relevant were included. A second literature review was performed after multiple online meetings held for identifying the consensus statements according to the population, intervention, comparison, and outcome (PICO) framework, when applicable for those statements discussing interventions. A final list of statements was agreed upon in May 2023 after three online meetings. The UEG framework has been followed [5].

### 2.3 Step 2: Modified Delphi Process

A multiple rounds modified Delphi process was applied for identifying the final statements/recommendations to be included in the consensus. More specifically, all statements were transferred onto a Google form, and each participant voted on the level of agreement. The level of agreement was assessed on a 6‐point likert scale, namely agree strongly (A+), agree with minor reservation (A), agree with major reservation (A−), disagree with minor reservation (D), disagree with major reservation (D−), and strongly disagree (D+). Although a specific cut‐off of agreement was not set for approving the final statement, all those statements having more than 10% disagreement (D, D− or D+) were discussed with the whole group, amended and re‐voted by all participants in a second and third round of voting. After the second or third round of voting, in all cases, a final agreement of > 80% was reached.

### 2.4 Step 3: Final Statements and Brief Explanatory Text

Each final statement was agreed upon by all the participants. Each statement is followed by a brief explanatory text. Since most of our statements are either definitions of malabsorption, expert‐based recommendations, or good clinical practice recommendations, we could not fully apply the GRADE methodology for rating the quality of evidence. This is also because, in the very minority of statements regarding tests or interventions, either the quality of evidence was overall poor, or other dedicated, recent guidelines or consensus already exist.

The present consensus has been split into two parts, in order to enhance its readability and to better focus on specific issues around this topic.

## 3 Definitions, clinical phenotypes, and diagnostic criteria

### 3.1 What Are the Definitions of Maldigestion and Malabsorption?

#### 3.1.1 Statement

Maldigestion is defined as the defective hydrolysis of large‐molecular nutrients into absorbable small molecular components. Malabsorption is defined as an impaired ability to absorb nutrients through the intestinal mucosa into the blood stream or lymphatic vessels. In the clinical context, the sole use of the term ‘malabsorption’ is acceptable.

According to our statement, the term maldigestion refers to the altered mechanisms of digestion of the ingested food, while the term malassimilation combines both maldigestion and malabsorption. The clinical presentation and complications of maldigestion and malabsorption are similar. Therefore, for clinical purposes, the distinction between malabsorption and maldigestion is of limited value [6]. Mechanisms that determine malabsorption can be divided into premucosal abnormalities (or flawed intraluminal digestion), mucosal abnormalities (maldigestion or malabsorption), mucosal intracellular or postcellular, and vascular and lymphatic transport abnormalities (malabsorption) [6].

#### 3.1.2 Statement

The malabsorption syndrome, that is, the clinical symptoms and signs caused by malabsorption, is determined by the type and amount of the malabsorbed substrates, by the deficiency of the malabsorbed nutrients, by the underlying disease, and by the consequences of accumulation of the malabsorbed substrate in the lumen of the gastrointestinal tract.

The metabolic and absorptive capacity of the colon and its microbiome may be clinically relevant for providing nutrients for absorption and for causing symptoms and complications of malabsorption. Colonic bacterial metabolism of carbohydrates to short chain fatty acids, which are readily absorbable in the colon [7, 8], results in the salvage of calories [9]. However, colonic salvage of malabsorbed nutrients can also result in symptoms and complications such as renal stones resulting from colonic hyperabsorption of oxalate [10] with consequent hyperoxaluria or symptoms because of the accumulation of gases resulting from bacterial metabolism of malabsorbed carbohydrates [11, 12]. The healthy colon has the capacity to absorb a wide variety of substances and nutrients including sodium, chloride, water, short‐chain fatty acids, calcium, magnesium, water‐soluble vitamins (biotin, folate, pantothenic acid [B5], pyridoxine [B6], riboflavin [B2], thiamine [B1]), and vitamin K [13, 14].

Malabsorptive symptoms caused by deficiency of nutrients are, among others, anaemia, weight loss, oedema, bleeding, osteomalacia, and sexual dysfunction. Malabsorptive symptoms caused by excess substrate in the intestine are, among others, steatorrhoea, diarrhoea, bloating, abdominal pain, and kidney stones [6].

### 3.2 Which Clinical Phenotypes Underlie Malabsorption?

#### 3.2.1 Statement

Malabsorption may be congenital (e.g., mucosal carrier proteins defects), secondary to surgical procedures or caused by many diseases or conditions originating from, or affecting, the small intestine, the pancreas, the liver, the biliary tract and the stomach. Pathophysiological mechanisms that cause malabsorption can be divided into premucosal abnormalities, mucosal abnormalities, and mucosal intracellular/postcellular and vascular transport abnormalities.

Malabsorption may be the result of congenital (primary) defects of mucosal carrier proteins. These defects may clinically result in a large variety of abdominal and extra‐abdominal symptoms and organ complications, including dermatologic, neurologic and psychiatric manifestations and mental retardation [6]. Malabsorption can be caused by non‐congenital (secondary) conditions, namely surgical procedures and by many diseases originating from or involving the small intestine and the pancreas, the liver, biliary tract and stomach [6]. In clinical practice, pathophysiological mechanisms may be mixed and several organs may be affected.

Physiologic processes other than digestion and absorption contribute to the normal absorption of nutrients, vitamins, and minerals. Solubilization is a prerequisite for absorption of nutrients such as fat or calcium [6]. Fat and fat‐soluble vitamins are solubilized by the formation of micelles, and calcium is solubilized through acidification in the gastrointestinal lumen. Alternatively, increased solubilization of the components of intestinal chyme may contribute to the manifestations of gastrointestinal diseases, such as increased absorption of oxalate, which can result in the development of kidney stones [10]. Liberation of substrate [6] (e.g., vitamin B12) from binding sites in food or, conversely, binding to proteins such as the intrinsic factor allows its absorption. Chemical changes to nutrients [15] may be required for absorption, such as reducing the charge of iron from Fe^+++^ to Fe^++^. Intestinal sensory and motor function permits detection of the presence of nutrients, facilitates adequate mixing of nutrients with intestinal secretions and delivery to absorptive sites, and provides adequate time for nutrient absorption. Neural and hormonal functions are also required to stimulate and coordinate digestive secretions, mucosal absorption, and intestinal motility [16].

#### 3.2.2 Statement

Malabsorptive disorders are characterised by a wide clinical spectrum, ranging from subtle clinical presentations to clinically obvious malabsorption symptoms, or to clinical presentations dominated by extra‐intestinal manifestations.

In some malabsorptive diseases, symptoms and clinical consequences of malabsorption may be the presenting clinical features, whereas in other diseases, the consequences of malabsorption may be obscured by more prominent symptoms of the underlying disease. The symptoms and clinical consequences of malabsorption may become clinically evident only after an extended duration of malabsorption. Malabsorptive disorders, may have subtle clinical presentations or mainly extraintestinal manifestations [6]. Typical malabsorption symptoms are weight loss, malnutrition or failure to thrive, dehydration, steatorrhoea, and chronic diarrhoea. Other manifestations in which malabsorption should be considered as the underlyeing cause are hypoproteinemia, anaemia, electrolyte imbalances, abdominal symptoms such as bloating, change in bowel habits and abdominal pain. Indeed, many clinical conditions that result in malabsorption are characterised by chronic diarrhoea, and this may mimick some functional bowel disorders including functional diarrhoea and irritable bowel syndrome. In this regard, a recent guideline on functional bowel disorders with diarrhoea has been released by the UEG [17]. In this guideline, although the authors recommend a symptom‐based approach as compared with a diagnostic strategy of exclusion in patients with chronic diarrhoea, they also suggest further testing when organic disorders are suspected, and in selected cases, in addition to full blood count, C reactive protein, CD serology, and faecal calprotectin in all cases.

Malabsorption and its clinical presentation may be restricted to deficiency of single nutrients and may present clinically as specific deficiency disorders, such as hypocalcemia, resulting in osteomalacia and osteoporosis, or iron or vitamin B12 deficiency [18, 19] resulting in blood cell count alterations (including isolated anisocytosis or mean cell volume alterations, anaemia, thrombocytopaenia or thrombocytosis, leucopenia). Sometimes extra‐abdominal symptoms may become prominent such as menstrual disturbances, growth delay in children, neurological alterations, miscarriage, or infertility [6].

### 3.3 What Are the Most Appropriate Diagnostic Criteria for Malabsorption?

#### 3.3.1 Statement

Clinical history, physical examination and routine laboratory tests may be helpful in raising the suspicion of malabsorption, selecting specific tests to document deficiencies and their sequelae, and detecting the underlying disease. Moreover, the presence of malabsorption should be considered in patients with known gastrointestinal, pancreatic, liver and biliary diseases.

The presence of malabsorption should be considered in all patients with symptoms of gastrointestinal, pancreatic and biliary diseases. The challenges in the management of malabsorption are to suspect that malabsorption is present, diagnose the cause of malabsorption and treat the underlying disease, detect and treat nutritional deficiencies and ease gastrointestinal and extra‐gastrointestinal symptoms.

Malabsorption is usually suspected on the basis of the patient's history, signs and symptoms, or findings on routine laboratory evaluations. A list of first‐line examinations for initial assessment of malabsorption can be found in the algorithm of Part 2 of the consensus.

#### 3.3.2 Statement

There is no universal diagnostic testing for malabsorption. The diagnosis of malabsorption can be made in case of clinical and laboratory evidence of micro‐ or macronutrient deficiency in a patient who has an adequate eating habit and no increased nutritional needs explaining this condition, and in case of evidence of a medical or surgical condition leading to malabsorption.

Malabsorption of some substrates or nutrients can be confirmed by measuring their increased stool concentration or decreased serum concentration or urinary excretion. Finding the cause of malabsorption often requires tests such as endoscopy with small intestinal biopsies; under certain clinical circumstances, non‐invasive tests or radiologic imaging are helpful in providing a specific diagnosis (such as abdominal ultrasound, computer tomography or magnetic resonance for the diagnosis of inflammatory bowel disease or chronic pancreatitis). Clinical history, physical examination and routine laboratory tests may be helpful in raising the suspicion of malabsorption, in selecting specific tests to document deficiencies and their sequelae, and in detecting the underlying disease. Clinical clues or results of laboratory tests can indicate the presence of a specific underlying disease or can help in the differential diagnosis. Approaches may differ depending on the travel history, and epidemiologic or ethnic background of an individual patient. Previous surgical procedures should alert the potential presence of reduced digestive or absorptive capacities, or their complications such as small intestinal bacterial overgrowth (SIBO).

## 4 Initial assessment, testing for malabsorption and its causes

### 4.1 How Should Physicians Establish a Diagnosis of Malabsorption?

#### 4.1.1 Statement

Tests and exams should be ordered depending on clinical data, medical and surgical history, and family history. Recognizing the cause of malabsorption often requires several examinations such as endoscopy with small intestinal biopsies, non‐invasive tests evaluating gastrointestinal function or radiological imaging.

The long list of differential diagnoses of malabsorption (Table 2) requires a systematic approach based on thorough history (family, medical, surgical or drug use), clinical examination, and laboratory results (see 3.3.2). Macronutrient malabsorption may lead to protein‐energy malnutrition [20], while micronutrient deficiency may lead to specific clinical presentations (Table 3). Isolated carbohydrate malabsorption often leads to digestive symptoms such as bloating, flatulence, abdominal pain, nausea or diarrhoea [12, 21]. The next step in explorations will often involve the digestive tract, that is, stool assessment (steatorrhoea, faecal elastase, ova and parasites), breath tests, duodenal biopsies and CT or MR enterography [22]. Guidelines or recommendations for specific tests, diseases or clinical situations exist, such as for the use of breath tests [23, 24], intestinal failure [25] or seronegative villous atrophy [26], and should be followed accordingly.

**TABLE 2:** Aetiologies of malabsorption according to the physio‐pathological mechanism involved.

Premucosal abnormalities	Mucosal abnormalities	Mucosal intracellular/postcellular and vascular transport abnormalities
Exocrine pancreatic insufficiency (chronic pancreatitis/pancreatic resection/cystic fibrosis/pancreatic adenocarcinoma) Cholestasis and malabsorption of bile salts (chronic cholestasis or bile leak/extensive ileal resection/partial or total gastrectomy) Small intestinal bacterial overgrowth Disaccharidase deficiency	Intestinal failure/deficiency ◦ Small bowel resection ◦ High output fistula ◦ Extensive enteropathy Coeliac disease Tropical sprue Diffuse small intestinal lymphoma Crohn's disease Infectious: Giardiasis, anguillosis, cyclosporiasis, cryptosporidiosis, Whipple's disease, HIV, tuberculosis, tropical sprue, small intestinal bacterial overgrowth Iatrogenic: Angiotensin II type 2 receptor blockers, NSAIDs, azathioprine, mycophenolate, methotrexate, graft‐versus‐host disease, chemotherapy, immunotherapy, radiation enteritis Eosinophilic gastroenteritis Mastocytosis Autoimmune enteropathy Common variable immunodeficiency Amyloidosis Autoimmune atrophic gastritis	Obstruction of lymphatic drainage, lymphangectasia Disorders causing rupture of the epithelial barrier with or without loss of mucosal substance Mesenteric ischaemia

**TABLE 3:** Main manifestations of specific micronutrient (vitamins or minerals) deficiency.

Micronutrient	Associated manifestations
Vitamin A	Xerophthalmia, dry mucous membranes, corneal ulcers, impaired night vision
Vitamin D	Rickets, osteoporosis
Vitamin E	Haemolytic anaemia, neuropathy, myelopathy
Vitamin K	Haemorrhagic syndrome, ecchymosis
Vitamin B1	Beriberi, Gayet‐Wernicke encephalopathy
Vitamin B2	Seborrhoeic dermatitis, cheilitis, angular stomatitis, glossitis
Vitamin B3 (PP)	Pellagra, rash, diarrhoea, dementia
Vitamin B6	Skin lesions (seborrhoeic, acne), neuro‐psychiatric, haematological (microcytic anaemia)
Vitamin B8	Dermatitis, alopecia
Vitamin B9 (folic acid)	Megaloblastic anaemia, pancytopenia, neurological (apathy, headache, insomnia), gastrointestinal (diarrhoea, abdominal pain), infertility, foetal abnormalities
Vitamin B12	Megaloblastic anaemia, pancytopenia, subacute combined degeneration of the spinal cord, neuro‐psychiatric alterations, glossitis, infertility/miscarriage, foetal abnormalities, hyper‐homocysteinaemia
Vitamin C	Scurvy, delayed wound healing, bruising
Chrome	Peripheral neuropathy, ataxia, diabetes
Copper	Hypochromic anaemia, cardiac arrhythmia, neutropenia, ataxia
Iron	Anaemia, thrombocytosis, asthenia, taste alterations, growth retardation
Fluoride	Osteoporosis, dental caries
Iodine	Hypothyroidism, goitre
Selenium	Cardiomyopathy (keshan disease), hypothyroidism
Zinc	Ageusia, alopecia, rash, immune deficiency, diarrhoea

### 4.2 Which Tests Need to Be Performed for Identifying Malabsorption and Its Causes?

#### 4.2.1 Statement

Malabsorption of some ingested nutrients or substrates can be confirmed by measuring their increased stool concentration, or their decreased serum or urine concentration or urinary excretion.

Measuring the concentration of various substrates in stool samples could serve as a useful marker of malabsorption. Quantitative determination of faecal fat involves measuring the excretion of fat in grams from a patient who has consumed a standardized amount of dietary fat, typically 100 g [27]. This method was originally introduced into clinical practice to diagnose malabsorption in patients with exocrine pancreatic insufficiency (EPI) [28]. Despite its long‐standing and valuable use, its clinical value has become limited. This method quantifies steatorrhoea; however, it is not designed to differentiate between various causes of malabsorption (e.g., pancreatic, enteric, or biliary). Additionally, its practical application in clinical settings is challenging because it involves 72‐h stool collection and handling of faecal samples. Therefore, its utility is often restricted to cases where other diagnostic methods yield inconclusive results [29, 30, 31]. Measurement of the faecal steatocrit, that is, faecal fat excretion in a random spot stool, has been suggested as an alternative for semiquantitative assessment of faecal fat excretion [32].

Faecal pancreatic elastase‐1, a proteolytic enzyme secreted by the pancreas, serves as a specific marker for EPI [33]. More details will be provided in 4.2.5 statement.

Faecal chymotrypsin, a protease produced by the pancreas, serves as another surrogate marker, the reduction of which can be utilized for detecting advanced chronic pancreatitis. However, this method is not widely available, and has been replaced by pancreatic elastase‐1 [34].

The increased concentration of alpha‐1 antitrypsin in stool samples may indicate protein‐losing enteropathy. Alpha‐1 antitrypsin is a glycoprotein that resists degradation by digestive enzymes and is employed as a marker to detect the presence of proteins in the intestinal lumen, where its presence is consistent with protein‐losing enteropathy [35, 36].

Several methods for the detection of bile acid malabsorption have been used, such as the faecal concentration of bile acids, and the ^14^C cholylglycine test. However, due to the limitations of these tests, and with the availability of alternatives such as SeHCAT testing, which exhibits high sensitivity and specificity, these methods are rarely used nowadays [37]. More recently, the measurement of serum concentrations of C4 and fibroblast growth factor (FGF)‐19 has also been proposed as a promising alternative [38]. The detection of urinary 5‐hydroxyindoleacetic acid (HIAA), a serotonin metabolite, is primarily focussed on the detection of neuroendocrine tumours as they can increase serotonin levels. However, there is evidence suggesting that urinary 5‐HIAA levels may sometimes be elevated in intestinal malabsorption, such as in CD [39, 40]. Finally, faecal calprotectin is a non‐invasive method which is useful for rasing the suspicion of intestinal inflammation [41].

The D‐xylose test will be discussed in a dedicated statement (4.2.3). A more comprehensive diagnostic algorithm for the diagnosis of malabsorption in adults and children can be found in Part 2 of the consensus.

#### 4.2.2 Statement

In case of clinical suspicion of malabsorption with no apparent cause, coeliac disease serology should be assessed. Oesophago‐gastro‐duodenoscopy with duodenal biopsies, and in selected cases small bowel capsule endoscopy, and ileo‐colonoscopy with biopsies should be considered to detect mucosal diseases explaining the malabsorption.

In patients presenting with malabsorption dominated by diarrhoea, steatorrhoea, weight loss or failure to thrive without an apparent cause, CD must be suspected [42].

Identification of tissue transglutaminase 2 antibodies (TGA) as the target antigen for anti‐endomysium antibodies (EMA) allows reliable and inexpensive screening for the disease [43]. IgA‐EMA is part of the serological diagnosis of CD. In adults, the IgA antideamidated gliadin peptide antibody assay (anti‐DGP) can be a substitute for IgA‐TGA if this assay is not available [44]. IgG‐based assays (TG2 or DGP) are indicated for identifying CD in patients with selective IgA‐deficiency [45]. Therefore, total IgA levels need to be measured concurrently with serology testing.

According to available guidelines, diagnosis of CD must be confirmed by intestinal biopsy in adults [46] which differs from that in children, where the European Society for Paediatric Gastroenterology, Hepatology and Nutrition (ESPGHAN) guidelines allow for a no‐biopsy approach in selected patients with high titres of TGA ( > 10 times upper limit of normal) [47]. However, more recent evidence from prospective studies [48, 49], as well as a systematic review [50], support the no‐biopsy approach in selected adult cases. More in depth, adult patients having TGA > 10 times upper limit of normal and a moderate to high pre‐test probability of CD could be diagnosed without duodenal biopsy. Indeed, more evidence on this, as well as future guidelines, are awaited before such approach could be extensively applied. It should be considered that, in adults, a serology‐only approach would not allow the diagnosis of patients with potential, refractory, and seronegative CD [51, 52].

Endoscopic features of CD differ, with one‐third of patients having normal endoscopic appearance [53], indicating the need for intestinal biopsies [54]. Endoscopic alterations, such as duodenal scalloping and Kerckring's folds flattening [55], must always prompt duodenal biopsies, especially in patients who had not been suspected of having CD. The mucosal changes of the duodenum in CD are traditionally classified according to Marsh‐Oberhuber et al. [56] or Corazza‐Villanacci [57].

Assessment of intestinal architecture can help in differentiating other types of enteropathy [58] and allows for disaccharidase assays, although this procedure is mostly confined to the research setting [59]. Causes of enteropathies and their main features are reported in Table 1. Endoscopy with biopsy is also indicated for diagnostic evaluations that require both endoscopic and histological evaluations, such as in Crohn's disease [60].

The main limitation of the oesophagogastroduodenoscopy is its inability to reach beyond the duodenum, which can be overcome, when required in exceptional clinical circumstances such as in the case of complicated CD (i.e., enteropathy‐associated T‐cell lymphoma, ulcerative jejuno‐ileitis and refractory CD), by double balloon enteroscopy, and to some extent by video capsule endoscopy, which offers visual assessment without the possibility to obtain biopsies [61]. It has been suggested that, when complicated CD is suspected, video capsule endoscopy could be used as a first‐line examination for discriminating patients deserving a double balloon enteroscopy [62, 63]. This sequential approach yields a good accuracy in this setting.

#### 4.2.3 Statement

D‐xylose testing has been used in the past to assess small bowel mucosa absorptive function, but its clinical usefulness is limited.

D‐xylose is an actively absorbed and passively diffusing five‐carbon monosaccharide that has been used for many years to assess the intestinal absorption capacity. Its absorption is similar to that of glucose and its elimination is mostly renal and mostly in a non‐transformed form. Information on its accuracy derives from few and dated studies that mostly focussed on patients with CD. According to a seminal review, in adults, the standard protocol is based on the ingestion of 25‐g D‐xylose in 250–600 mL of water in the fasting state, with an analysis of a 5‐h urine collection and a 1‐h serum sample, discriminating between normal subjects and patients with proximal small intestinal malabsorption with a high specificity and sensitivity ( > 95%). In paediatric patients, the 1‐h serum test after administration of 5 g of D‐xylose was also highly sensitive ( < 91%) and specific (nearly 100%) [64].

However, liver diseases with ascites, dehydration, gastroparesis, renal insufficiency, and SIBO may result in a false‐positive test. In order to avoid the consequences of an impaired renal function, especially in older adults, authors have suggested to use priority 1‐h blood results and correct them to a standardised body surface area of 1.73 m^2^ (xylose levels x actual surface area/1.73) [65], but others favour urine measurements [65, 66]. Other changes regarding doses ingested and time points for blood and urine collection were suggested but were not studied on a large scale nor are controlled trials available. ^14^C‐xylose and ^13^C‐xylose breath tests and H_2_ breath tests with D‐xylose were evaluated in the diagnosis of malabsorption in CD patients [67] but with a varying sensitivity and only in one study.

In summary, because of the small number of studies, with a small sample size and potential bias, and because of the scarce availability of the substrate, this test is not currently indicated in the setting of malabsorption.

Conversely, d‐xylose may be useful in some limited clinical settings and in tertiary referral centres, such as in the follow‐up of intestinal failure patients after small bowel transplant or during GLP‐2 analogue treatment [68].

#### 4.2.4 Statement

The currently available evidence does not support the use of breath testing for diagnosing small intestinal bacterial overgrowth.

Breath tests have long been used to assess carbohydrate malabsorption (see Part 2 of the consensus) and SIBO [59, 69, 70, 71, 72, 73]. In practical terms, the patient is given a carbohydrate to ingest, and several quantitative measurements of exhaled hydrogen (H_2_) and methane (CH_4_), as well as symptoms experienced, are taken at regular intervals. Carbohydrate malabsorption results in colonic fermentation of the ingested and/or unabsorbed substrate. Exhaled H_2_ and/or CH_4_ are only produced by fermentation and thus reflect the arrival of the unabsorbed carbohydrate in the colon. SIBO will result in fermentation of the ingested sugar in the small intestine by enteric bacteria that should not be present at that level [74, 75, 76, 77, 78, 79, 80, 81, 82, 83, 84, 85].

The most recent guidelines [17, 86] and expert consensus [87] have discouraged the use of breath tests for the diagnosis of SIBO due to several limitations affecting their accuracy. For example, breath test results for SIBO do not correlate with symptoms or small bowel aspirates [88], and do not predict response to antibiotics. According to the latest meta‐analysis, the lactulose breath test has a pooled sensitivity of 42% and pooled specificity of 70.6% [89]. For the glucose breath test only, in patients with a predisposing condition to SIBO (i.e., history of abdominal surgery), the accuracy was higher (sensitivity 81.7% and specificity 78.8%) but still suboptimal. All this would translate into a high rate of false positive tests with subsequent risk of unnecessary antibiotic treatment. It has also been suggested to associate a test of oro‐caecal transit time to improve breath test accuracy for SIBO [23]; however, even those tests are burdened by high variability and poor performance [90, 91], or have been abandoned in clinical practice (e.g., scintigraphy).

SIBO can be asymptomatic or result in many clinical manifestations, from simple abdominal symptoms such as bloating, abdominal discomfort, flatulence, diarrhoea, and unspecific symptoms such as fatigue and poor concentration to more severe manifestations such as malabsorption, nutrient deficiency and weight loss [92, 93, 94, 95, 96, 97, 98]. SIBO should only be considered in clinical practice, in the presence of predisposing conditions, such as anatomical abnormalities or post‐surgery structural gut changes (e.g., gastric bypass or Roux‐en‐Y), medications or conditions slowing down gut motility, small bowel dysmotility and other diseases such as hepatic encephalopathy, gastroparesis, Parkinson's disease, chronic pancreatitis and end‐stage renal disease [72, 75, 99]. To date, there is no consensus on a universal test for detecting SIBO. Culture of jejunal aspirates used to be considered as a gold standard [88] but this procedure is invasive, difficult to perform and, in certain circumstances, may lack sensitivity [72, 100, 101, 102, 103, 104, 105]. Our knowledge in the field of gut microbiota, motility, and functional disorders is rapidly evolving [91], and the concepts of SIBO and small bowel dysbiosis, along with their diagnosis, are likely to change in the near future.

#### 4.2.5 Statement

We suggest the use of the ^13^C‐mixed triglyceride breath test or steatocrit to measure fat malabsorption when available. In patients with suspected steatorrhoea, measurement of faecal elastase or the ^13^C‐mixed triglyceride breath test may be used to detect EPI.

Fat malabsorption is a typical result of severe EPI but can also be due to other causes including untreated CD, diseases affecting the terminal ileum, and short bowel syndrome. Under physiological circumstances, no more than 7% of fat ingested on a high fat diet (100 g/d) is excreted in the faeces, and measuring the quantitative amount of faecal fat is the reference method for estimation of fat malabsorption [106, 107]. During recent years, the ^13^C‐mixed triglyceride breath test has been established as an alternative to quantitative faecal fat measurements [24]. It is based on the principle that digestion and absorption of triglycerides will ultimately lead to ^13^CO_2_ production in the liver and that the amount of ^13^CO_2_ exhaled after ingestion of a labelled test meal correlates with intestinal lipid absorption. Since intestinal lipolysis by pancreatic lipase is a prerequisite for lipid absorption, test results have also been shown to correlate with pancreatic lipase secretion. Direct comparisons with the reference standard (determination of pancreatic enzyme and/or bicarbonate output in duodenal aspirates following exogenous stimulation) demonstrate 90%–100% sensitivity for moderate to severe EPI with specificity ranging between 80% and 90% in adults [108, 109, 110]. Repetitive ^13^C‐mixed triglyceride breath tests in patients with EPI before and after initiation of pancreatic enzyme therapy showed that this test can also be used for monitoring therapeutic efficacy [111, 112, 113, 114]. Despite these obvious advantages, the availability of the test is still limited.

By contrast, as mentioned before, measurement of faecal elastase concentration is widely available and additionally or primarily recommended by gastroenterology societies for the diagnosis of EPI [115, 116]. Elastases are stable during gastrointestinal transit and in faecal samples. Measurement requires a random stool sample and is usually performed using an ELISA specific for the human enzyme. Concentrations of less than 200 microgram per gram of stool are considered pathological. However, while many studies have shown a negative correlation between faecal elastase concentration and the probability of EPI, no clear cut‐off has been established. Thus, the recently published AGA Clinical Practice Update on EPI advises that a faecal elastase level < 100 μg/g of stool provides good evidence of EPI, while levels of 100–200 μg/g are indeterminate [116]. Moreover, meta‐analyses have shown that even in cohorts with a high pre‐test probability of EPI, there is a 10% false negative rate, and in those with a low pre–test probability, the false‐positive rate is 11% [33]. Thus, normal faecal elastase concentrations cannot exclude EPI and have limited accuracy for differential diagnosis of chronic diarrhoea.

#### 4.2.6 Statement

CFTR gene study and chloride sweat testing should be conducted in young patients with EPI and suspicion of cystic fibrosis.

The vast majority of patients (85%–90%) with cystic fibrosis will present with EPI leading to severe malabsorption at birth or in infancy [117]. Cystic fibrosis is the most important differential diagnosis in children with EPI. Diagnosis in individuals outside of newborn screening relies on the combination of clinical evidence, for example, EPI, and evidence of CF transmembrane conductance regulator (CFTR) dysfunction [118]. The latter can be demonstrated using chloride sweat testing, CFTR molecular genetic analysis, or *CFTR* physiologic tests. Guidelines on diagnosis of cystic fibrosis recommend that a chloride sweat test should be performed first, followed by other tests in equivocal cases [119].

## Author Contributions

M.V.L. and A.D.S. proposed the writing of the consensus to the UEG; they drafted the protocol, involved all scientific societies, and coordinated all the steps. All authors participated in all the steps, including literature review, first draft of the statements, voting, and writing of each statement. H.F.H., I.T., R.B., S.S., and A.F. acted as group leaders. M.V.L. wrote the final manuscript, collated and reviewed all statements, and critically reviewed the whole consensus. G.R.C. and A.D.S. critically reviewed the consensus and provided supervision through all the steps. All authors revised and approved the final version of the manuscript.

## Ethics Statement

The authors have nothing to report.

## Consent

The authors have nothing to report.

## Conflicts of Interest

The authors declare no conflicts of interest.

## Permission to Reproduce Material From Other Sources

Not applicable.

## Disclaimer

This consensus has been developed with reasonable care and with the best of knowledge available to the authors at the time of preparation. They are intended to assist healthcare professionals and allied healthcare professionals as an educational tool in providing information that may support them in providing care to patients. Patients or other community members using this consensus should do so only after consultation with a health professional and should not mistake this consensus as professional medical advice. This consensus must not substitute seeking professional medical and health advice from a health professional. This consensus may not apply to all situations and should be interpreted in the light of specific clinical situations and resource availability. It is up to every clinician to adapt this consensus to local regulations and to each patient's individual circumstances and needs. The information in this consensus shall not be relied upon as being complete, current or accurate, nor shall it be considered as inclusive of all proper treatments or methods of care or as a legal standard of care. UEG makes no warranty, express or implied, in respect of this consensus and cannot be held liable for any damages resulting from the application of this consensus, in particular for any loss or damage (whether direct or indirect) resulting from a treatment based on the guidance given herein. UEG shall not be held liable to the utmost extent permissible according to the applicable laws for any content available on such external websites, which can be accessed by using the links included herein.

## Supporting information

Supporting Information S1

Supporting Information S2

Supporting Information S3

Supporting Information S4

Supporting Information S5

Supporting Information S6

## Data Availability

All data generated for developing this consensus have been included in this paper. No other data are available.
