# Supplementary material for: European Consensus on Malabsorption—UEG & SIGE, LGA, SPG, SRGH, CGS, ESPCG, EAGEN, ESPEN, and ESPGHAN. Part 1: Definitions, Clinical Phenotypes, and Diagnostic Testing for Malabsorption
Source: United European Gastroenterol J. 2025 Mar 25;13(4):599–613. doi: 10.1002/ueg2.70012 (PMC12090837; doi:10.1002/ueg2.70012)
Supplement: Supplementary file 1 — Supporting Information S1 [file UEG2-13-599-s003.docx]

Search for the question **Which high-risk groups should be screened for malabsorption?**

There were 2 strings that were searched

1. (("Population Characteristics"[Mesh])) AND (("Malabsorption Syndromes"[Mesh]))

In **PubMed** this search returned 3710 titles.

We applied the following limits to this search : clinical study, guidelines, meta-analysis, multicenter study, observational study, practice guidelines, systematic review + Humans, and articles in English, and the studies included and screened were 340

In **EMBASE**, with the same key words – without filters – 686 titles, with filters/limits – 406 titles - which were finally screened

**EMBASE:**

'population characteristics'/exp AND 'malabsorption syndromes'/exp – 686

#1 AND ('case control study'/de OR 'clinical article'/de OR 'clinical study'/de OR 'cohort analysis'/de OR 'comparative study'/de OR 'cross sectional study'/de OR 'human'/de OR 'longitudinal study'/de OR 'medical record review'/de OR 'multicenter study'/de OR 'observational study'/de OR 'practice guideline'/de OR 'prospective study'/de OR 'retrospective study'/de) AND ('article'/it OR 'article in press'/it) = 406

1. The second search combined the first search with the most common symptoms in malabsorption.

In **PubMed**, we searched in all fields, using Mesh terms:

(("Population Characteristics"[Mesh]) OR ("Population Characteristics"[All Fields])) AND (("Malabsorption Syndromes"[Mesh]) OR ("Malabsorption Syndromes"[All Fields])) OR (("Malabsorption Syndromes"[Mesh]) OR ("Malabsorption Syndromes"[All Fields])) AND (("Osteoporosis"[Mesh]) OR ("Osteoporosis"[All Fields]) OR ("Diarrhea"[Mesh]) OR ("Diarrhea"[All Fields]) OR ("Steatorrhea"[Mesh]) OR ("Steatorrhea"[All Fields]) OR ("Failure to Thrive"[Mesh]) OR ("Failure to Thrive"[All Fields]) OR ("Anemia"[Mesh]) OR ("Anemia"[All Fields]) OR ("Weight Loss"[Mesh]) OR ("Weight Loss"[All Fields])) - 7603 titles

We applied the following limits to this search: clinical study, guidelines, meta-analysis, multicenter study, observational study, practice guidelines, systematic review + Humans, and articles in English, and the studies included and screened were 338

In **EMBASE:**

#1 AND #2 AND #3 = 166

#3

('malabsorption syndromes'/exp OR 'malabsorption syndromes') AND ('osteoporosis'/exp OR 'osteoporosis' OR 'diarrhea'/exp OR 'diarrhea' OR 'steatorrhea'/exp OR 'steatorrhea' OR 'failure to thrive'/exp OR 'failure to thrive' OR 'anemia'/exp OR 'anemia' OR 'weight loss'/exp OR 'weight loss') = 29,843 titles

#2

('population characteristics'/exp OR 'population characteristics') AND ('malabsorption syndromes'/exp OR 'malabsorption syndromes') = 699 titles

#1

('case control study'/de OR 'clinical article'/de OR 'clinical study'/de OR 'cohort analysis'/de OR 'comparative study'/de OR 'cross sectional study'/de OR 'human'/de OR 'longitudinal study'/de OR 'medical record review'/de OR 'multicenter study'/de OR 'observational study'/de OR 'practice guideline'/de OR 'prospective study'/de OR 'retrospective study'/de) AND ('article'/it OR 'article in press'/it)

**Identification of studies via databases and registers**

Records removed *before screening*:

Records marked as ineligible by automation tools (n = 11578)

Records identified from:

Databases (n =12698)

**Identification**

Records excluded* (n = )

Duplicates=192

Irrelevant = 361+350=711

Records screened

(n = 1120)

Reports sought for retrieval

(n = 99+118=217

Reports not retrieved

(n = ….)

No full text available n=3+

**Screening**

Reports assessed for eligibility

(n = 96+118=214

Reports excluded:

Other languages (n = 12+1)

Review (n =+7 )

Small sample size (n = 6+5)

Diagnosis based on symptoms n=2

Dg based on Ab, n= 4

Other subject, n=20

This would be file 2.

Studies included in review

(n = 76+81=157)

**Included**

*If automation tools were used, indicate how many records were excluded by a human and how many were excluded by automation tools.
