# Supplementary material for: European Consensus on Malabsorption—UEG & SIGE, LGA, SPG, SRGH, CGS, ESPCG, EAGEN, ESPEN, and ESPGHAN. Part 1: Definitions, Clinical Phenotypes, and Diagnostic Testing for Malabsorption
Source: United European Gastroenterol J. 2025 Mar 25;13(4):599–613. doi: 10.1002/ueg2.70012 (PMC12090837; doi:10.1002/ueg2.70012)
Supplement: Supplementary file 3 — Supporting Information S3 [file UEG2-13-599-s005.docx]

Pubmed and EMBASE search for MeSH (malabsorption) AND (elderly) OR (pregnancy)

**Identification of studies via databases and registers**

Records removed *before screening*:

Duplicate records removed (n =205 )

Records marked as ineligible by automation tools (n =0 )

Records removed for other reasons (n = 0)

Records identified from:

Databases (n =3108)

**Identification**

Records screened

(n =2903)

Records excluded*

(n =0 )

Reports sought for retrieval

(n =2903)

Reports not retrieved

(n = 0)

**Screening**

Reports assessed for eligibility

(n = 2903)

Reports excluded:

Reason 1: title not relevant (n =2694)

Reason 2: abstract not relevant (n=60)

Reason 3

etc.

Studies included in review

(n =149)

**Included**

*If automation tools were used, indicate how many records were excluded by a human and how many were excluded by automation tools.

*From:*  Page MJ, McKenzie JE, Bossuyt PM, Boutron I, Hoffmann TC, Mulrow CD, et al. The PRISMA 2020 statement: an updated guideline for reporting systematic reviews. BMJ 2021;372:n71. doi: 10.1136/bmj.n71
